# Levels and trends of adolescent girl’s undernutrition and anemia in West and Central Africa from 1998 to 2017

**DOI:** 10.7189/jogh.11.13006

**Published:** 2021-08-10

**Authors:** Anne-Sophie Le Dain, Vera Sagalova, Roger Sodjinou, El Hassane Tou, John Ntambi, Sebastian Vollmer, Noel Marie Zagre

**Affiliations:** 1 United Nations Children’s Fund, West and Central Africa Regional Office, Dakar, Senegal; 2 Heidelberg Institute of Global Health, University of Heidelberg, Heidelberg, Germany; 3 United Nations Children’s Fund, Niamey, Niger; 4 Department of Economics and Centre for Modern Indian Studies, University of Goettingen, Goettingen, Germany; 5 UNICEF Area Representative for Gabon and São Tomé and Príncipe and to the ECCAS, Libreville, Gabon

## Abstract

**Background:**

Adolescence is a highly vulnerable period of human life characterized by substantial physiological and cognitive changes for which adequate nutrition is crucial. To date, evidence on determinants, prevalence, and trends of undernutrition and anemia for the entire West and Central African region is missing. This paper provides evidence on trends and levels of adolescent anemia and undernutrition in West and Central Africa.

**Methods:**

We pooled all Demographic and Health Surveys (DHS) for West and Central African countries that were conducted between 1986 and 2017 to analyze levels and trends of adolescent anemia and undernutrition. We investigated the association of adolescent undernutrition and anemia within this region with World Bank income level classification of the country.

**Results:**

Our findings suggest that the regional prevalence of adolescent anemia and undernutrition remained high at 45% and 19% respectively over the last 20 years. Anemia increased in about one third of countries and undernutrition in about two thirds over the studied period. On the aggregated level, these trends are largely masked and both levels remained stable in the entire region between the years 1998 and 2017. The results of the multivariable regression analysis indicate an association of adolescence with undernutrition and anemia, which was independent of socio-economic factors such as income, education, and place of residence.

**Conclusion:**

We conclude that levels of adolescent undernutrition and anemia remain high with little progress over the last 20 years and that adolescence is a significant correlate of both anemia and undernutrition. Given the recognition of the international community that adolescent nutrition is an important public health concern in resource-poor settings, there is an urgent need to improve data availability, quality, and use for decision-making and to design successful high-impact interventions to combat adolescent malnutrition in low- and middle-income countries.

The number of adolescents (aged 10-19 years) reached a historical maximum of 1.8 billion in the year 2010 with 90% living in low- and middle-income countries [1]. In 2015, roughly one million adolescents are estimated to have died prematurely. The leading causes of death among 15-19 age group were HIV/AIDs, road injuries, self-harm, and interpersonal violence [1]. This age group is usually neglected from public health interventions as adolescents naturally suffer less morbidity and mortality compared to other age-groups [1]. As a result, most health interventions are designed to address leading causes of death, neglecting the nutritional needs of adolescents [1]. However, adolescence is a highly vulnerable period of human life characterized by substantial physiological and cognitive changes for which adequate nutrition is crucial [2].

Sustainable Development Goal number 2 sets out to eliminate all forms of malnutrition by 2030 [3]. Available empirical literature to date provides estimates of anemia and undernutrition prevalence mostly on individual country level. Some studies provide evidence on determinants and prevalence of anemia on global and country level [4-8], and some studies on undernutrition on country level [1,2,8,9]. To date, evidence on determinants, prevalence and trends of undernutrition and anemia for the entire West and Central African subregion is missing. In recognition of this problem UNICEF commissioned the present work of conducting a secondary data analysis of DHS to take stock of adolescent nutrition in the region.

Various papers estimate anemia prevalence for individual countries within this subregion: eg, anemia prevalence among non-pregnant, non-lactating women including adolescent girls aged 15 to 19 years is estimated at 63% in Gabon, 51.7% in Côte d’Ivoire, 54.5% in Republic of the Congo, 55.7% in Senegal, 58.2% in Gambia, 62% in Burkina Faso and 42% in Benin while it is 72.5% in Burkina Faso, 67.9% in Gambia, 64.9% in Guinea, 64.1% in Togo, 63.6% in Côte d'Ivoire and 61.4% in Senegal among pregnant adolescents [5,10,11]. The prevalence of underweight and stunting is estimated at 6.4% and 5.4% among adolescents in Nigeria, respectively [1]. Due to differences in methodology, underlying surveys, as well as survey periods, such single-country estimates are often difficult to compare.

This paper contributes to existing literature by providing evidence on anemia and undernutrition prevalence in adolescent girls and their trends over roughly 20 years in the entire West and Central African region. This time span is applicable for select countries only, for some countries and variables of interest the first and last data points represent a shorter period of time.

## METHODS

### Data

We pooled all Demographic and Health Surveys (DHS) for West and Central Africa countries (according to UNICEF regional definition) that were conducted between 1986 and 2017. For most analyses, we considered either only the most recent available survey or only phases 4-7 (corresponding to years 1998-2017). The sample includes adolescent girls (aged 15 to 19) and adult women (aged 20 to 49). The pooled sample contains information on 1 287 100 individuals. Biometric and anthropometric data are only available for a subset of countries and within those, only for a subset of eligible women. Anemia information is provided for 169 664 women, of whom 36 773 are adolescents, and undernutrition information is available for 314 769 women and 62 644 adolescents. A detailed list of countries and survey years for each outcome is provided in **Table 1** and **Table 2**.

**Table 1:** Prevalence of adolescent girl’s anemia by country in West and Central Africa in first and most recent survey*

	First available survey				Most recent survey				
**Country**	**Year**	**Prevalence (%)**	**95% CI**	**N**	**Year**	**Prevalence (%)**	**95% CI**	**N**
Benin	2001	65.15	0.61-0.69	594	2011	41.56	0.38-0.46	886
Burkina Faso	2003	51.94	0.48-0.56	964	2010	47.94	0.45-0.51	1643
Cameroon	2004	45.61	0.42-0.49	1274	2011	39.66	0.37-0.43	1839
Congo	2005	56.45	0.52-0.61	720	2011	55.11	0.50-0.60	1125
Congo Dem. Rep.	2007	48.70	0.44-0.54	934	2013	40.13	0.36-0.44	1977
Cote d‘Ivoire	2011	53.86	0.50-0.58	961	2011	53.86	0.50-0.58	961
Gabon	2012	63.52	0.59-0.68	1203	2012	63.52	0.59-0.68	1203
Gambia	2013	56.08	0.52-0.61	1078	2013	56.08	0.52-0.61	1078
Ghana	2003	45.95	0.43-0.49	1022	2014	47.73	0.44-0.52	878
Guinea	2005	50.95	0.47-0.55	800	2012	47.06	0.43-0.51	1087
Mali	2001	62.16	0.58-0.67	726	2012	50.75	0.47-0.55	914
Niger	2006	46.72	0.42-0.51	839	2012	45.99	0.41-0.50	851
São Tomé and Príncipe	2008	51.39	0.45-0.58	518	2008	51.39	0.45-0.58	518
Senegal	2005	60.50	0.57-0.64	1112	2017	57.19	0.54-0.61	1894
Sierra Leone	2008	50.55	0.46-0.56	553	2013	49.46	0.46-0.53	1793
Togo	2013	54.73	0.51-0.58	902	2017	53.74	0.50-0.58	790

CI – confidence interval

*In Cote d’Ivoire, Gabon, The Gambia and São Tomé and Príncipe, anemia data are only available in one survey.

**Table 2:** Adolescent undernutrition - prevalence by country in West and Central Africa in first and most recent survey*

	First available survey				Most recent survey			
**Country**	**Year**	**Prevalence (%)**	**95% CI**	**N**	**Year**	**Prevalence (%)**	**95% CI**	**N**
Benin	1996	14.12	0.10-0.19	205	2011	12.19	0.11-0.14	2774
Burkina Faso	1992	14.50	0.10-0.19	299	2010	20.36	0.18-0.23	1617
Cameroon	1998	8.45	0.05-0.12	245	2011	8.52	0.06-0.11	1831
Central African Republic	1994	13.53	0.10-0.17	318	1994	13.53	0.10-0.17	318
Chad	1996	20.94	0.17-0.25	494	2014	22.23	0.20-0.24	2331
Congo	2005	18.68	0.15-0.22	1486	2011	21.72	0.18-0.26	1138
Congo Dem. Rep.	2007	23.04	0.17-0.29	935	2013	17.70	0.15-0.20	1938
Cote d'Ivoire	1994	6.75	0.04-0.09	497	2011	14.31	0.12-0.17	978
Gabon	2000	7.39	0.05-0.10	433	2012	15.32	0.12-0.19	1203
Gambia	2013	24.05	0.21-0.28	1034	2013	24.05	0.21-0.28	1034
Ghana	1993	10.71	0.06-0.16	140	2014	12.95	0.10-0.16	873
Guinea	1999	11.22	0.08-0.15	399	2012	16.52	0.14-0.19	1080
Liberia	2006	15.93	0.13-0.19	1287	2013	13.58	0.10-0.17	912
Mali	1995	15.87	0.13-0.19	593	2012	16.60	0.14-0.19	906
Niger	1992	19.53	0.15-0.24	386	2012	24.06	0.21-0.27	828
Nigeria	2003	21.65	0.19-0.25	1640	2013	19.71	0.18-0.21	7531
São Tomé and Príncipe	2008	12.88	0.08-0.17	480	2008	12.88	0.08-0.17	480
Senegal	1992	18.32	0.14-0.23	273	2010	29.78	0.26-0.34	1296
Sierra Leone	2008	12.51	0.09-0.16	556	2013	13.26	0.11-0.16	,807
Togo	1998	14.25	0.10-0.19	244	2013	10.78	0.09-0.13	895

CI – confidence interval

*In Central African Republic, The Gambia, and São Tomé and Príncipe, undernutrition data are only available at one point in time, hence no trends over time can be established within these countries.

### Outcomes

Outcome variables are indicator variables for undernutrition and anemia. Undernutrition is defined by a BMI of less than 18.5Kg/M^2^. Since undernutrition is difficult to measure in pregnant women, we exclude those currently pregnant from undernutrition analyses. Anemia is an indicator variable for any form of anemia including mild, moderate, and severe. Thus, a hemoglobin level of less than 12g/dl for non-pregnant women and below 11g/dl for pregnant women denotes anemia in our study (these cut-offs are provided by the DHS program and their levels are already adjusted for altitude).

### Exposure

The main exposure is a binary indicator for being adolescent, ie, being between 15 and 19 years of age. While our sample contains some (few) data points from adolescents below age 15, biometric and anthropometric data are only available for those 15 and older. Furthermore, younger adolescents (10-14) are not part of the sampling design of the surveys and are hence excluded from these analyses.

Control variables include basic socio-economic factors such as educational attainment, wealth quintile, and rural/urban residence. Some specifications also include primary sampling unit fixed effects to control for factors that are specific to the local environment, either physical or social, where women live.

### Statistical analysis

All descriptive analyses were weighted with the DHS survey weights to account for over- or under-sampling of certain clusters, which were then rescaled with the population size of the individual countries, so that all composite indicators represent the population makeup of the West and Central African region. All regressions were linear probability models. For each outcome we ran a simple linear regression with an indicator variable for adolescent girls as main explanatory variable. In a second specification we added all covariates and in a third specification we additionally included primary sampling unit fixed effects.

## RESULTS

**Figure 1:**
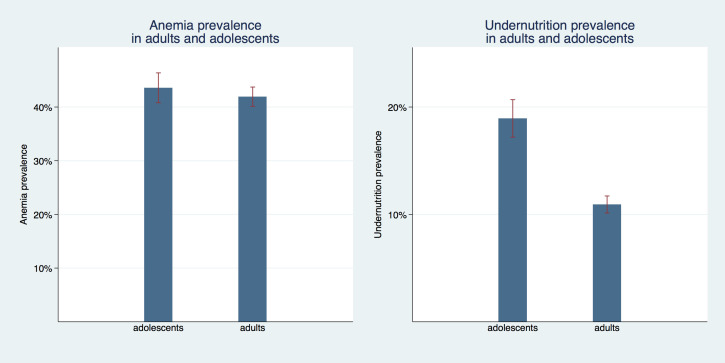
Anemia and undernutrition prevalence in adult women and adolescent girls in West and Central Africa between 1986 and 2017. Note: Anemia defined as any anemia of any degree (mild, moderate, or severe).

**Figure 1** compares the undernutrition and anemia prevalence of adolescent girls and adult women. For undernutrition we see a sizable difference between these age groups with a prevalence of 10.9% (95% confidence interval (CI) = 10.1%-11.7%) for adults compared to 19.0% (95% CI = 17.2%-20.7%) for adolescents. Prevalence of anemia was not statistically different between adults with 41.9% (95% CI = 40.1%-43.7%) and adolescents with 43.5% (95% CI = 40.8%-46.3%).

**Figure 2:**
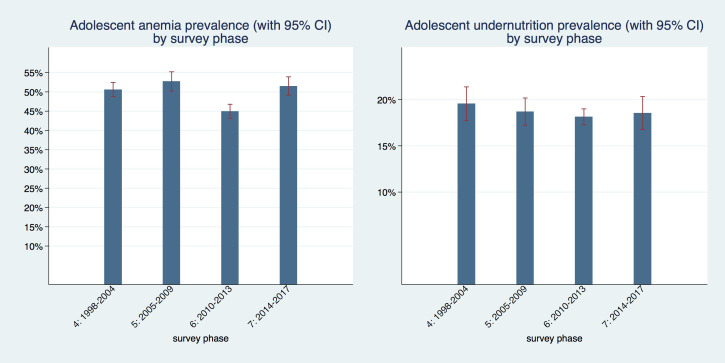
Anemia and undernutrition prevalence in adult women and adolescent girls by survey phase in West and Central Africa.

In **Figure 2** we show the prevalence of adolescent undernutrition and anemia over time organized by DHS phases. The coverage of countries differs marginally from phase to phase; therefore, trends have to be interpreted with some caution. Coverage of countries in each phase is shown in Table S1 for undernutrition and Table S2 for anemia, in the **Online Supplementary Document**. For undernutrition, prevalence rates in all four time periods were not statistically different from each other, they ranged from 18.1%to 19.6 percent, but all confidence intervals overlap to some extent. For anemia, prevalence was slightly above 50% in phases four (1998-2004), five (2005-2009) and seven (2014-2017) with overlapping confidence intervals. Phase six (2010-2013) had the lowest prevalence with 44.9% (95% CI = 43.1%-46.7%), which was also statistically significantly different from the other three phases. It is also the phase with the most comprehensive coverage of countries in the region and therefore likely provides the best estimate for the region as a whole.

**Figure 3:**
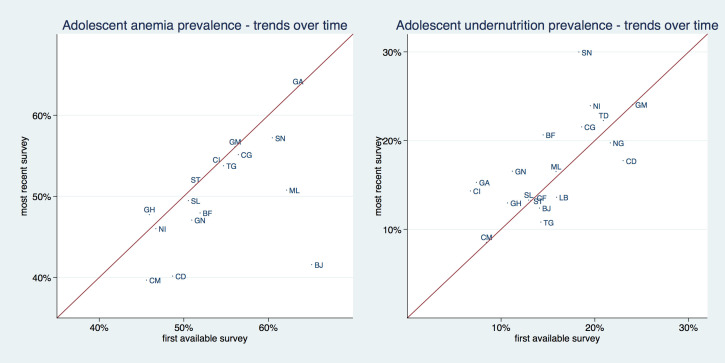
Prevalence of adolescent girl’s undernutrition and anemia by country comparing the first and last available survey.

**Figure 3**, and **Table 1** and **Table 2** show the prevalence of adolescent undernutrition and anemia by country comparing the first and last available survey. The solid line in **Figure 3** is a 45° line, the x-axis shows the prevalence for the first available and the y-axis for the most recent survey. Countries that are situated above the 45° line increased their respective prevalence and countries that are situated below the 45° line decreased it. The distance from the 45°-degree line indicates the magnitude of the increase or reduction in prevalence. Undernutrition increased in roughly two thirds of investigated countries. Anemia increased in only about one third of surveyed countries, and only to a small extent, bringing the data points barely above the 45° line, while it decreased in two thirds of countries, mostly to a larger extent. Senegal had the highest undernutrition prevalence with 29.8% and Cameroon had the lowest with 8.5%. Gabon had the highest anemia prevalence with about 63.5% while Cameroon had the lowest with 39.7%. Benin was the country with the largest absolute reduction in anemia, going from 65.2% to 41.6%. Senegal had the highest absolute increase in undernutrition, going from 18.3% to 29.8%.

**Figure 4:**
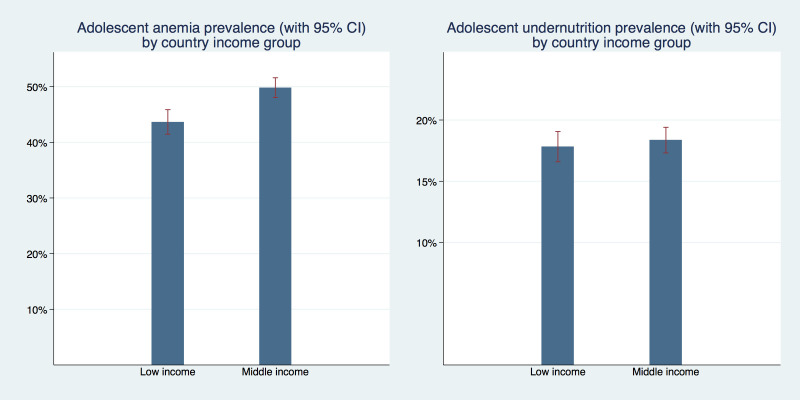
Prevalence of adolescent girl’s undernutrition and anemia separately for low- and middle-income countries in West and Central Africa.

In **Figure 4** we display the prevalence of adolescent undernutrition and anemia separately for low- and middle-income countries. The undernutrition prevalence was not statistically significantly different with 17.8% (95% CI = 16.6%-19.0%) in low-income and 18.4% (95% CI = 17.3%-19.4%) in middle-income countries. Anemia prevalence was higher in middle-income countries with 49.8% (95% CI = 48.0%-51.5%) compared to 43.6% (95% CI = 41.4%-45.9%) in low-income countries. This difference was statistically significant.

**Table 3** provides the results of various regression models investigating the difference in undernutrition and anemia prevalence between adolescents and adults. Columns 1 and 4 report the unadjusted correlations. Columns 2 and 5 show the differences adjusted for select socio-economic variables and the results provided in Column 3 and 6 are additionally adjusted for primary sampling unit fixed effects (PSU FE). The unadjusted correlations show that for both outcomes the prevalence is almost one percentage point higher in adolescents compared to adults. This is a relatively small difference which is also relatively robust to adjustments for socio-economic variables or primary sampling unit fixed effects.

**Table 3:** Linear regression models investigating the difference in undernutrition and anemia prevalence between adolescents and adults

	(1)	(2)	(3)	(4)	(5)	(6)
**Outcome:**	**Anemia**	**Anemia**	**Anemia**	**Undernutrition**	**Undernutrition**	**Undernutrition**
Adolescent	0.00947‡ (0.003)	0.0157§ (0.003)	0.0158§ (0.003)	0.0839§ (0.002)	0.0931§ (0.002)	0.0933§ (0.002)
Reference: less than primary education						
Primary or incomplete secondary		-0.0233§ (0.003)	-0.0217§ (0.003)		-0.0311§ (0.002)	-0.0290§ (0.002)
Secondary or higher		-0.0454§ (0.007)	-0.0451§ (0.007)		-0.0363§ (0.002)	-0.0305§ (0.002)
Reference: first quintile						
Second quintile		-0.00134 (0.005)	-0.000717 (0.005)		-0.0155§ (0.002)	-0.0142§ (0.002)
Middle quintile		-0.0161§ (0.005)	-0.0149‡ (0.005)		-0.0211§ (0.003)	-0.0188§ (0.003)
Fourth quintile		-0.0391§ (0.005)	-0.0377§ (0.005)		-0.0307§ (0.003)	-0.0283§ (0.003)
Richest quintile		-0.0605§ (0.006)	-0.0577§ (0.006)		-0.0430§ (0.003)	-0.0399§ (0.003)
Reference: rural:						
Urban		0.0123‡ (0.005)	0.0104† (0.005)		-0.0119§ (0.002)	-0.0163§ (0.002)
PSU fixed effects	No	No	Yes	No	No	Yes
Constant	0.506§ (0.002)	0.527§ (0.003)	0.526§ (0.003)	0.0920§ (0.001)	0.130§ (0.002)	0.129§ (0.002)
Observations	169 664	162 833	162 833	314 769	287 938	287 938

CI – confidence interval, PSU – primary sampling unit

*Standard errors in parentheses.

†*P* < 0.05.

‡*P* < 0.01.

§*P* < 0.001.

## DISCUSSION

We found that undernutrition levels differ significantly between adolescent girls and adult women in West and Central Africa Region, with adolescence accounting for roughly 9 percentage points of the difference. At the same time, adolescence was associated with only a minor (but significant) increase in probability of being anemic in the region and anemia prevalence was only marginally higher for adolescent girls than for adult women. Simultaneously, both indicators developed differently over time: while anemia prevalence has fallen in most of the individual countries, undernutrition prevalence has risen in roughly two thirds of the study countries. For the purpose of direct comparison of nutritional status between older adolescents and adult women, we employ Body Mass Index (BMI) with a uniform cut-off of BMI < 18.5 kg/m^2^ for undernutrition definition, thus probably slightly overestimating the true undernutrition prevalence. However, in the context of early marriage and pregnancy [12,13], physical demands imposed on these adolescent girls are similar to those adult women face, and we thus believe that in this context it is appropriate to use the same standard for adolescent girls and adults.

One interesting approach to address this measurement issue is suggested by Jeyakumar et al. who propose defining appropriate Mid-Upper-Arm circumference (MUAC) cut-offs for adolescent girls and validate their claim with an empirical assessment of correlation between results obtained through BMI and MUAC measurements [14]. A request to add such a measurement to standard DHS anthropometry has been already voiced by the research community but not implemented by the DHS program so far. Our results on trends in undernutrition are also difficult to compare with existing literature: only very few studies focus on trends in adolescent undernutrition and those that do employ a different methodology. Jaacks et al. provide the only recent comprehensive study on this topic that we could locate, however, the authors compute recent trends, expressed as annualized change between the two most recent surveys [15]. Within this time frame they report an overall reduction of adolescent undernutrition in the entire Sub-Saharan African region. However, they also report high regional heterogeneity in trends and find an increase in adolescent undernutrition (stratified by urban/rural location) for Niger, Guinea, Burkina Faso, Cote d’Ivoire, Ghana, Congo, Gabon, Mali, and Senegal – so roughly in half of West and Central African countries for which DHS data are available, and this list is consistent with our findings on long-term trends in the region [15].

One important drawback of this (and any other DHS-based) study is that no biometric or biomarker data are systematically collected on younger adolescent population (10-14) and all available data focusses on women, while adolescent boys are currently neglected by these surveys. An expansion of DHS (and potentially MICS surveys, which in their current form, while focusing on similar issues at large, do not collect biometrics or biomarkers of adults) to both younger and male adolescents would provide the necessary basis to fill this knowledge gap.

## CONCLUSION

We conclude that levels of adolescent undernutrition and anemia remain high with little progress over the last 20 years and that adolescence is a significant correlate of both anemia and undernutrition. Given the consensus within the international community that adolescent nutrition is an important public health concern in resource-poor settings, more than ever so in the COVID-19 context, there is an urgent need to improve data availability, quality, and use for decision-making processes. It is imperative to design successful high-impact interventions to combat adolescent malnutrition in low- and middle-income countries, based on lessons learned and best practices from front-runner countries in the region, such as Ghana or Nigeria. Additionally, regional economic communities (RECs) and their member states are required to assign higher priority to adolescent health and nutrition programmes.

## Additional material


Online Supplementary Document
