# Supplementary material for: Levels and trends of adolescent girl’s undernutrition and anemia in West and Central Africa from 1998 to 2017
Source: J Glob Health. 2021 Aug 10;11:13006. doi: 10.7189/jogh.11.13006 (PMC8397282; doi:10.7189/jogh.11.13006)
Supplement: Online Supplementary Document [file jogh-11-13006-s001.pdf]

Table A1: Overview of available data: anemia prevalence in adolescent in West and Central Africa

	Survey phase				
Country	4	5	6	7	Total
Benin	3,124	5,969	5,513	0	14,606
Burkina Faso	4,211	0	8,525	0	12,736
Cameroon	5,137	0	7,819	0	12,956
Congo	0	3,502	5,768	0	9,27
Congo Dem. Rep.	0	4,632	9,328	0	13,96
Cote d'Ivoire	0	0	4,678	0	4,678
Gabon	0	0	5,645	0	5,645
Gambia	0	0	4,558	0	4,558
Ghana	5,691	4,916	0	4,704	15,311
Guinea	0	3,881	4,691	0	8,572
Mali	3,694	4,738	5,433	0	13,865
Niger	0	4,681	5,42	0	10,101
Sao Tome and Principe	0	2,615	0	0	2,615
Senegal	0	11,007	6,161	8,013	25,181
Sierra Leone	0	3,724	8,228	0	11,952
Togo	0	0	4,802	4,563	9,365
Total	21,857	49,665	86,569	17,28	175,371

Table A2: Overview of available data: undernutrition prevalence in adolescent girls in West and Central Africa

	Survey phase				
Country	4	5	6	7	Total
Benin	6,066	16,419	15,818	0	38,303
Burkina Faso	11,945	0	8,318	0	20,263
Cameroon	5,09	0	7,693	0	12,783
Chad	3,522	0	0	11,019	14,541
Congo	0	6,719	5,563	0	12,282
Congo, Dem. Rep.	0	4,621	9,216	0	13,837
Cote d'Ivoire	0	0	4,751	0	4,751
Gabon	2,77	0	5,334	0	8,104
Gambia	0	0	4,402	0	4,402
Ghana	7,525	4,704	0	4,595	16,824
Guinea	3,766	3,909	4,64	0	12,315
Liberia	0	6,818	4,519	0	11,337
Mali	11,918	13,989	5,159	0	31,066
Niger	0	4,442	4,997	0	9,439
Nigeria	7,271	31,632	37,435	0	76,338
Sao Tome and Principe	0	2,312	0	0	2,312
Senegal	0	4,44	5,553	0	9,993
Sierra	0	3,33	7,836	0	11,166
Togo	0	0	4,713	0	4,713
Total	59,873	103,335	135,947	15,614	314,769
